# Establishing a yeast-based screening system for discovery of human GLUT5 inhibitors and activators

**DOI:** 10.1038/s41598-017-06262-4

**Published:** 2017-07-24

**Authors:** Joanna Tripp, Christine Essl, Cristina V. Iancu, Eckhard Boles, Jun-yong Choe, Mislav Oreb

**Affiliations:** 1 Institute of Molecular Biosciences, Goethe University, Max-von-Laue Straße 9, 60438 Frankfurt am Main, Germany; 2 Department of Biochemistry and Molecular Biology, Rosalind Franklin University of Medicine and Science, The Chicago Medical School, 3333 Green Bay Road, North Chicago, IL 60064 USA

## Abstract

Human GLUT5 is a fructose-specific transporter in the glucose transporter family (GLUT, SLC2 gene family). Its substrate-specificity and tissue-specific expression make it a promising target for treatment of diabetes, metabolic syndrome and cancer, but few GLUT5 inhibitors are known. To identify and characterize potential GLUT5 ligands, we developed a whole-cell system based on a yeast strain deficient in fructose uptake, in which GLUT5 transport activity is associated with cell growth in fructose-based media or assayed by fructose uptake in whole cells. The former method is convenient for high-throughput screening of potential GLUT5 inhibitors and activators, while the latter enables detailed kinetic characterization of identified GLUT5 ligands. We show that functional expression of GLUT5 in yeast requires mutations at specific positions of the transporter sequence. The mutated proteins exhibit kinetic properties similar to the wild-type transporter and are inhibited by established GLUT5 inhibitors N-[4-(methylsulfonyl)-2-nitrophenyl]-1,3-benzodioxol-5-amine (MSNBA) and (−)-epicatechin-gallate (ECG). Thus, this system has the potential to greatly accelerate the discovery of compounds that modulate the fructose transport activity of GLUT5.

## Introduction

Most glucose transporters (GLUTs), members of the SLC2 family, facilitate the passive diffusion of glucose and related monosaccharides in mammalian cells. In humans there are 14 GLUTs, which differ in tissue distribution, primary sequence, substrate specificity and affinity in accordance with physiological needs^1, 2^. Unlike other GLUTs capable of fructose transport, GLUT5 is fructose-specific and does not transport glucose^3–5^. GLUT5 is expressed in intestine, kidney, sperm, fat and skeletal muscle cells^6^. High-fructose diet has been implicated in type II diabetes, hypertension, hyperuricemia, obesity, nonalcoholic fatty liver disease and increased risk of cardiovascular disease^4, 7–11^. As one of the major fructose transporters in humans, GLUT5 is an attractive therapeutic target in these diseases. For instance, in diabetic patients GLUT5 expression in muscle is significantly increased and drugs that enhance insulin action affect GLUT5 expression rate^12^. A recent study showed that GLUT5-mediated fructose absorption in the small intestine is enhanced through interaction of GLUT5 with the thioredoxin-interacting protein (Txnip; a protein that regulates glucose homeostasis), and in certain forms of diabetes Txnip expression and fructose absorption increase, suggesting a mechanism that links diabetes and the metabolic syndrome^13^. Cancer cells have higher demands for carbohydrate transport than normal cells and GLUT5 is upregulated in various cancers^14^. In pancreatic cancer cells, fructose metabolism is preferentially channeled to nucleic acid synthesis, potentiating cancer proliferation^15^. Increased use of fructose mediated by GLUT5 is a metabolic feature of acute myeloid leukemia (AML) and GLUT5 inhibition reduced the malignant leukemic phenotypes of AML cells^16^. Importantly, GLUT5 is normally absent in breast tissue but it is expressed in breast tumors^14^ and breast carcinoma cell lines MCF-7 and MDA-MB-231 have high levels of GLUT5 and fructose transport^17^. Given the medical importance of GLUT5, its inhibitors have the potential to become drugs for treatment of cancer or diabetes, however inhibitors of GLUT5 are scarce. They include natural product compounds that inhibit GLUT1 as well, like green tea catechins^18^ or Rubusoside (from *Rubus suavissimus*)^19^, but also GLUT5-specific inhibitors like Astragalin-6-glucoside (a glycosylated derivative of Astragalin, from *Phytolacca Americana*)^19^ and N-[4-(methylsulfonyl)-2-nitrophenyl]-1,3-benzodioxol-5-amine (MSNBA)^20^. The latter compound is the most potent and specific GLUT5 inhibitor reported so far.

Systems to assay GLUT5 transport activity include GLUT5 proteoliposomes^19, 20^, expression of GLUT5 in *Xenopus laevis* oocytes^21^, and human cell lines such as MCF-7^17^ or Caco-2^22^ cells. These systems require purified protein or labor-intensive and high-cost cell cultivation. Furthermore, analysis of GLUT5 in mammalian cells needs to take into account or eliminate interference from fructose transport by other GLUT proteins. Thus, establishing a microbial system without endogenous fructose transporters would be highly desirable to simplify the assaying of GLUT5 activity.

The yeast *Saccharomyces cerevisiae* is not only widely used for research of fundamental processes in a eukaryotic cell, but has also proved useful for functional studies on heterologous proteins as well as for high-throughput screening approaches, many of which have medicinal relevance^23^. For instance, yeast was used as a model system to study the mechanisms of neurodegenerative diseases^24^ and cancer^25^. For the analysis of sugar transporters from various sources, yeast has proved an excellent model system. To this end, a strain was constructed, in which all genes encoding hexose transporters and other transporters with hexose uptake activity have been deleted^26^. The strain is designated as hexose transporter-deficient (*hxt* ^*0*^) strain, EBY.VW4000; it is not able to grow on media with glucose, fructose or mannose as the sole carbon source and grows only very slowly on galactose. For maintenance, the strain is routinely cultivated on maltose, a disaccharide that is taken up through the specialized maltose symporters encoded by the *MALx1* loci^27^. Thus, the *hxt* ^*0*^ strain offers an excellent opportunity to clone and characterize heterologous hexose transporters, e.g. from fungi^28^ or plants^29^ by replacing the function of endogenous transporters. However, the functional expression of mammalian glucose transporters in the *hxt* ^*0*^ background proved to be a non-trivial task. In initial trials, the human glucose transporters GLUT1 and GLUT4 did not confer growth of the *hxt* ^*0*^ strain on glucose^30, 31^. In a later approach, the complementation of the *hxt* ^*0*^ phenotype by GLUT1 and GLUT4 could be achieved by prolonged incubation on glucose-containing media or UV-mutagenesis of the transformed yeast cells^32^. By genetic analyses, this could be attributed to mutations either in the GLUT transporter sequence or in the genome of the yeast host. For example, GLUT1 was functional only if it contained certain mutations in the second transmembrane domain or when the *hxt* ^*0*^ strain acquired the *fgy1* mutation^32^. Once the functionality of GLUT1 and GLUT4 in yeast was established, it could be shown that they exhibit comparable properties as in the native environment concerning transport kinetics and inhibition by Cytochalasin B^32^. Encouraged by these successes, we aimed at establishing a functional expression of human GLUT5 in the *hxt* ^*0*^ strain in the present work.

We show that mutated versions of GLUT5 are functional in yeast, have kinetic parameters similar to those in the native environment^17^, and are inhibited by the GLUT5-specific inhibitor MSNBA^20^ as well as by (−)-epicatechin-gallate (ECG), which is also a GLUT1 inhibitor^18^. Furthermore, inhibitor effect on GLUT5 fructose transport in the *hxt* ^*0*^ strain, as indicated by inhibitor IC_50_ value, is similar to that determined for GLUT5 inhibition in the human breast cancer cells MCF-7 (in the case of MSNBA) and in GLUT5-expressing *Xenopus laevis* oocytes (in the case of ECG). Additionally, both MSNBA and ECG decrease cell growth of GLUT5-expressing *hxt* ^*0*^ strain in a dose-dependent manner. With GLUT5 expression in the *hxt* ^*0*^ strain, inhibitors or activators of GLUT5 can be determined with the radioactive fructose uptake assay or by cell growth in fructose-based media. The latter method makes this system amenable to facile and rapid high-throughput screening of GLUT5 ligands, thus accelerating GLUT5 effector discovery. Subsequent optimization of GLUT5 ligands as selective and potent *in vivo* chemical probes will allow for advancement of our understanding of GLUT5 biology, its role in human disease, and ultimately its pharmaceutical control.

## Results

### Cloning and expression of GLUT5 in the *hxt*^*0*^ strain

**Figure 1:**
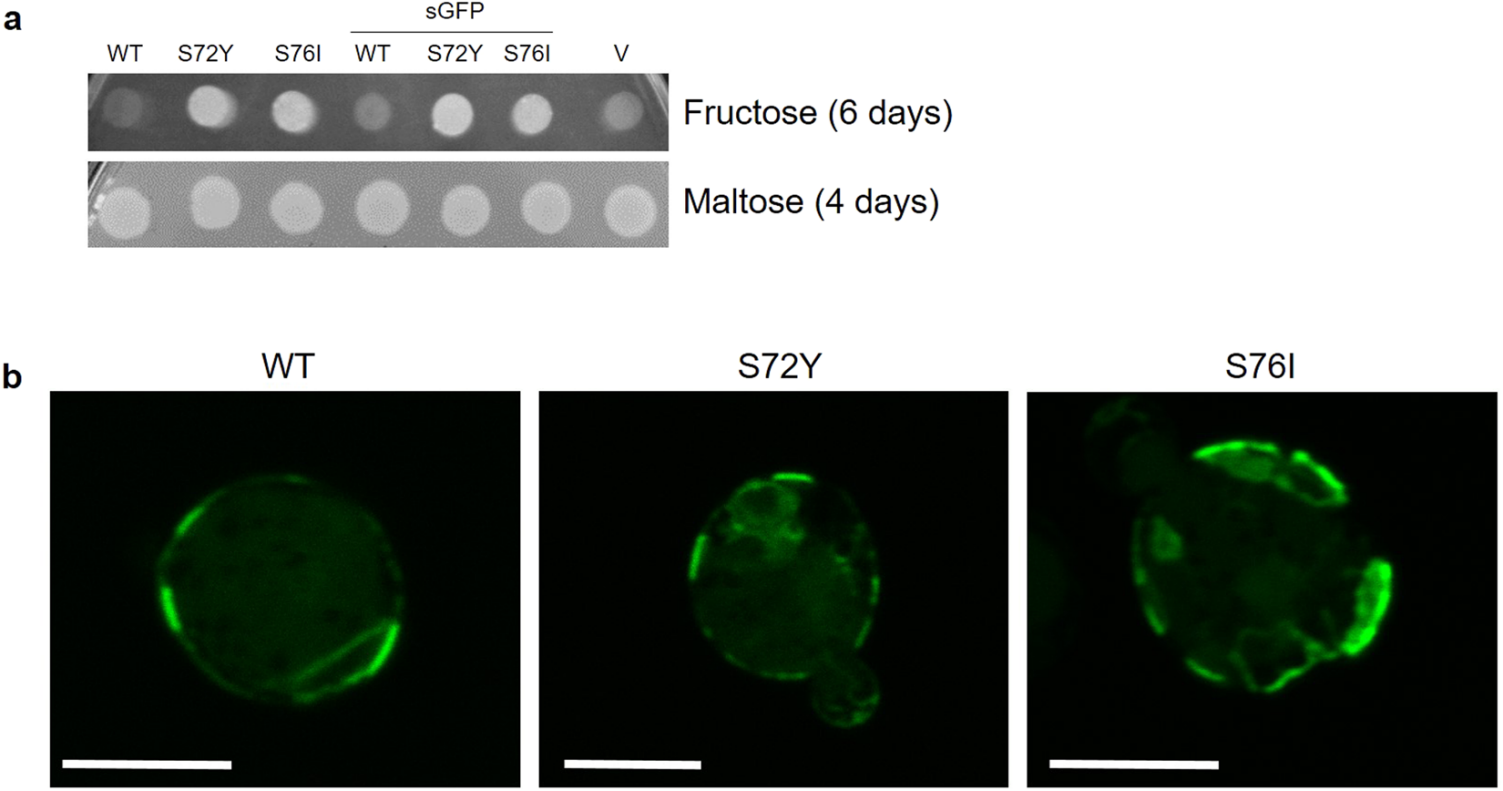
Functionality and subcellular distribution of GLUT5 variants. (**a**) Suspensions of EBY.VW4000 cells (OD_600nm_ = 1) transformed with plasmids encoding GLUT5tr, GLUT5tr^S72Y^, GLUT5tr^S76I^ or their fusions with sGFP were droped onto agar plates containing 2% (w/v) fructose (SCF-Ura-Met medium) or 1% (w/v) maltose (SCM-Ura medium). The latter medium was used to control the viability of the transformants and empty vector (V) was a negative control for growth on fructose. The duration of incubation at 30 °C is indicated. (**b**) The cells expressing the indicated variant of GLUT5-sGFP fusion proteins were cultivated in SCM-Ura-Met media until the exponential phase and analyzed by confocal laser scanning microscopy. Scale bar, 5 µm.

To generate plasmids for GLUT5 expression in yeast we used a construct previously codon-optimized for expression in insect cells and encoding a GLUT5 protein truncated for the first seven amino acids at the N-terminus (in the following referred to as GLUT5tr). The open reading frames were amplified using oligonucleotides with 30–40 basepair overhangs homologous to the *MET25* promotor and *CYC1* terminator of the p426MET25 vector^33^. A methionine-repressible promoter was chosen to minimize the risk of possible toxic effects due to overexpression of a heterologous membrane protein. In addition to GLUT5tr ORFs, a variant, in which the codons for the initial seven amino acids were re-introduced into GLUT5tr by an oligonucleotide (the resulting construct is designated as GLUT5 in the following), was also amplified. The PCR products were co-transformed with the linearized vector into EBY.VW4000 to allow for plasmid assembly by homologous recombination (“gap-repair” procedure)^34^. The transformants were plated on SCM-Ura medium to select for clones that have successfully assembled the plasmid. About 1000–3000 colonies were obtained on each plate. Subsequently, these clones were replica-plated onto SCF-Ura agar plates to test for the ability of GLUT5 to confer growth of yeast cells on fructose. On this medium, only a very low percentage of the clones could grow, suggesting that mutations might have occurred either in the GLUT5 sequence or in the genome of the *hxt* ^*0*^ strain, as previously observed with GLUT1 and GLUT4^32^. Therefore, we isolated and sequenced plasmids from the clones growing on fructose. Indeed, in GLUT5 ORFs of all isolated plasmids, single amino acid substitutions could be identified. In both GLUT5tr and GLUT5, the following mutations were isolated: S72Y (one clone of each GLUT5 and GLU5tr); S76I (three clones of each); S76N (one clone of GLUT5). Thus, in total, seven clones contained a mutation at position 76 and two at position 72. Strikingly, the positions S72 and S76 are directly adjacent to residues that align to positions W65 and V69 of GLUT1 (see alignment in the supplementary information, Supplementary Fig. S5). W65R and V69M mutations were found in GLUT1 variants, which were functional in yeast^32^. All these mutations are located in the second transmembrane helix (TM2) of GLUT5 or GLUT1. To test whether the mutations in the GLUT5 sequence alone are sufficient to confer growth of the *hxt* ^*0*^ strain on fructose, the plasmids were transformed into EBY.VW4000 again and growth was confirmed on fructose containing agar plates (for example, see Fig. 1a).

### Subcellular localization of GLUT5 variants in yeast cells

The mutations that promoted the functionality of GLUT5 in the *hxt* ^*0*^ strain could affect the intracellular trafficking of the protein. To investigate this possibility, we created constructs for C-terminal fusion of GLUT5tr, GLUT5tr^S72Y^ and GLUT5tr^S76I^ with superfolder GFP (sGFP)^35^. The functionality of the fusion proteins was demonstrated by growth test on fructose-containing agar plates (Fig. 1a). Subsequently, subcellular distribution of the fusion proteins was investigated by fluorescence microscopy. For all variants, an inhomogeneous distribution in the plasma membrane and a partial retention in the endomembrane systems could be observed (Fig. 1b). Thereby, we did not observe significant differences between the wild-type protein and the mutants, which indicates that the mutations do not directly affect the trafficking of the protein. Thus, some more subtle effects, such as the interactions between TM2 and lipids or the conformational flexibility of the transporter might be responsible for the functionality of the mutated protein in the yeast membrane. Similarly, the mutations in GLUT1 did not obviously affect the distribution of GLUT1 in subcellular fractions^32^.

### Structural modeling of GLUT5 mutants

**Figure 2:**
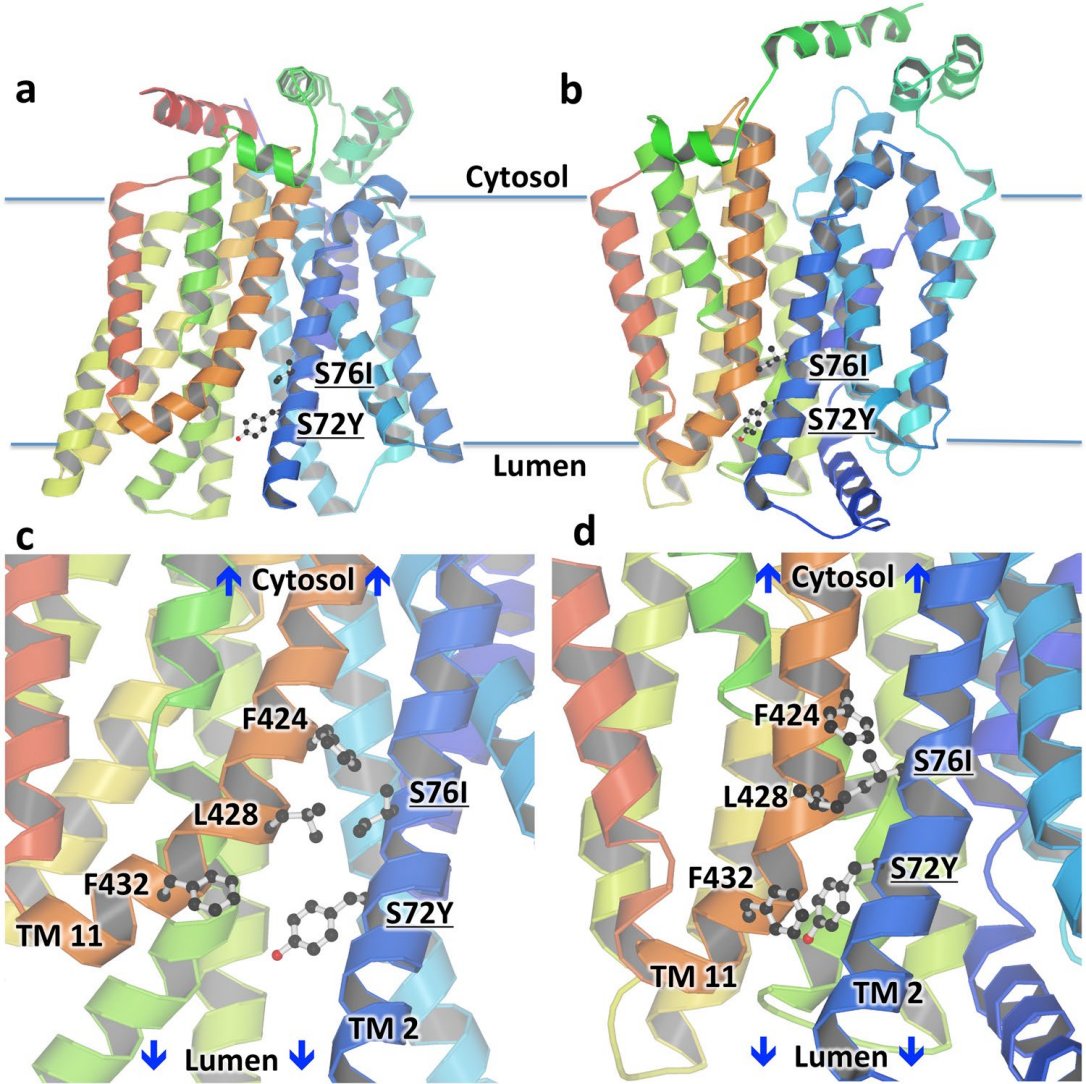
Structural models of GLUT5^S72Y^ and GLUT5^S76I^ mutants. Outward-facing (**a**) and inward-facing (**b**) structures of mutants. S72 and S76 are located toward the lumen in TM helix 2. Close-up showing the location of Y72 and I76 in the outward-facing (**c**) and inward-facing (**d**) conformations of the transporter. Y72 and I76 interact with hydrophobic residues from TM helix 11. Figure was generated with Molscript^47^ and Raster3D^48^.

As an approach to understand the effect of S72Y and S76I mutations on the molecular level, we performed structure modeling of GLUT5 mutants. GLUT5 is a Major Facilitator Superfamily (MFS) protein. Most MFS proteins have 12 transmembrane helices (TM) organized as two domains (the N- and C-terminal domains) related by a 2-fold pseudosymmetry axis. The substrate cavity is centrally housed between the two domains. The most widely accepted transport mechanism for MFS proteins is the alternating-access model^36^ also called “rocker-switch”, in which the substrate cavity is alternately opened to each side of membrane. Available crystal structures of MFS transporters are consistent with this model, showing the transporters in two major conformations: the so-called inward-facing (substrate cavity opened towards cytosol) and outward-facing (substrate cavity opened towards outside) conformations. Crystal structures of the mammalian GLUT5, both in the inward-facing and outward-facing conformations, were previously determined^37^. On the basis of these crystal structures, we generated the structural models for human GLUT5 in the two conformations to examine the potential effect of S72Y and S76I mutations on GLUT5 structure. Both residues S72 and S76 are in TM2 and interact with residues from TM11 (Fig. 2), specifically F424, L428 and F432. Sequence analysis of TM2 and TM11 for the residues in positions 72, 76, 424, 428 and 432, shows variable conservation of these residues (Supplementary Material, Fig. S5). In TM2, both S72 and S76 of GLUT5 tend to be conserved or replaced in other GLUTs by small residues like Ala or Gly. Mutations of these serines to bulkier residues, Tyr in position 72 or Ile in position 76, seem to be more consequential for the inward-facing conformation in which the space between TM2 and TM11 is tight and accommodation of the mutations requires repositioning of TM2 and TM11 (Fig. 2a and c and Table 1), compared with the wild-type structure; TM2 and TM11 push away from each other to fit the bulkier substitutions in position 72 or 76. In contrast, the outward-facing conformation has sufficient space between TM2 and TM11 to adopt the substitutions without any changes in the positions of TM2 and TM11, relative to those in the wild-type structure (Fig. 2b and d). The relative movement of TM2 and TM11 in GLUT5 S72Y and S76I outward-facing conformation structures suggests that S72Y substitution is more drastic than S76I in terms of surrounding space and interactions (Table 1).

**Table 1:** Movement of Cα atoms of TM helices 2 and 11 in GLUT5^S72Y^ or GLUT5^S76I^ relative to those in the wild-type, for the inward-facing conformation structures.

Residue position in TM2	S72Y (Å)	S76I (Å)	Residue position in TM11	S72Y (Å)	S76I (Å)
78	0.5	0.2	423	−0.2	−0.4
79	0.2	0.2	424	−0.2	−0.9
78	0.5	**0.2**	425	−0.3	−**0.9**
77	0.7	**0.4**	426	−0.3	−**0.4**
**76**	0.8	**0.4**	427	−0.3	−**0.7**
75	0.4	**0.2**	428	−0.4	−**1.3**
74	*1*.*8*	**0.2**	429	−*0*.*5*	−**1.2**
73	*1*.*7*	**0.5**	430	−*0*.*5*	−**0.5**
*72*	*1*.*6*	0.7	431	−*0*.*9*	−0.6
71	*0*.*4*	0.6	432	−*0*.*7*	−0.6
70	*0*.*9*	0.5	433	−*0*.*7*	−0.5
69	*1*.*4*	0.6	434	−*0*.*8*	−0.2
68	1.0	0.7	435	−0.9	−0.2
67	0.5	0.4	436	−0.9	−0.2

Average movements for 12 residues around the mutated residues (highlighted in italics for S72Y and bold for S76I) are 2.0 Å (for S72Y) or 1.1 Å (for S76I).

### Functional studies on GLUT5 mutants

**Figure 3:**
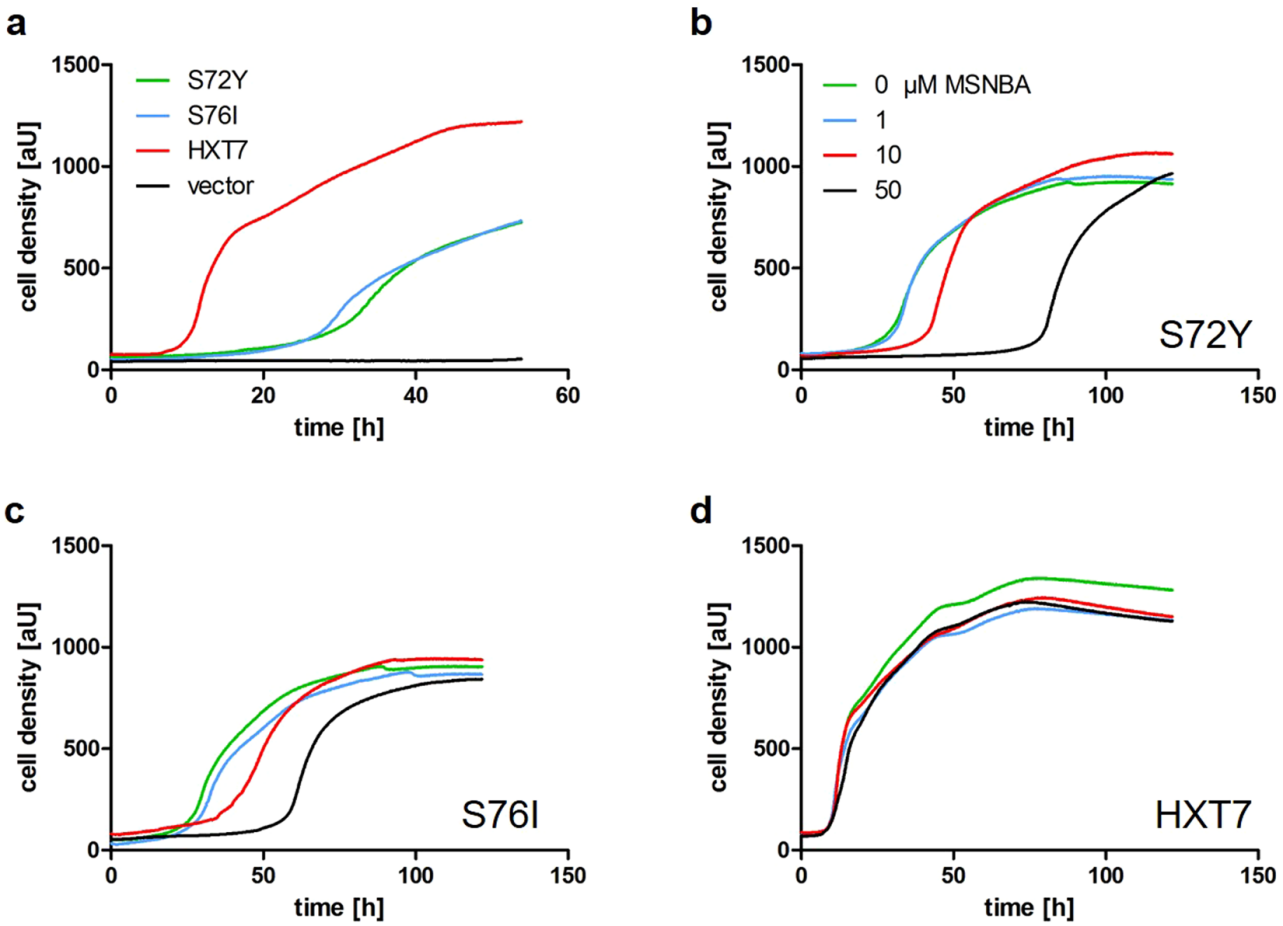
Growth analysis of GLUT5-transformed *hxt* ^*0*^ cells. (**a**) The EBY.VW4000 cells transformed with plasmids encoding GLUT5tr^S72Y^, GLUT5tr^S76I^ or Hxt7 were cultivated in YEP media containing 1% (w/v) fructose and 200 µg/ml of G418 for plasmid selection. The cells transformed with the empty vector were used as a negative control. The growth was measured using the Cell Growth Quantifier (Aquila Biolabs). (**b**–**d**) Indicated amount of MSNBA was added to the cultures and the growth was measured as in (a). The same color code is used throughout panels b–d.

Since GLUT5 conferred a rather slow growth on synthetic media (about five days until colonies appeared on agar plates; see Fig. 1a), we generated plasmids with dominant markers that can be maintained on rich media (YEP) for further functional studies. The ORFs of GLUT5tr, GLUT5tr^S72Y^ and GLUT5tr^S76I^ were placed under the control of the (constitutive) truncated *HXT7* promoter in the multicopy vector pRS72K. The growth of EBY.VW4000 transformed with these plasmids was first assessed on solid media (Supplementary Fig. S6). A plasmid encoding the endogenous hexose transporter Hxt7 capable of fructose transport^38^ and the empty vector were used as positive and negative controls, respectively. In line with the results obtained on synthetic media, only the mutated GLUT5 variants, but not the wild-type, could support growth on fructose. Next, we analyzed the growth of the transformants in liquid media using a Cell Growth Quantifier (Aquila Biolabs)^39^. Both GLUT5tr mutants showed significant growth in liquid media, albeit with a longer lag phase and slower rate compared to the transformants expressing the endogenous high-affinity hexose transporter *HXT7* (Fig. 3a).

**Figure 4:**
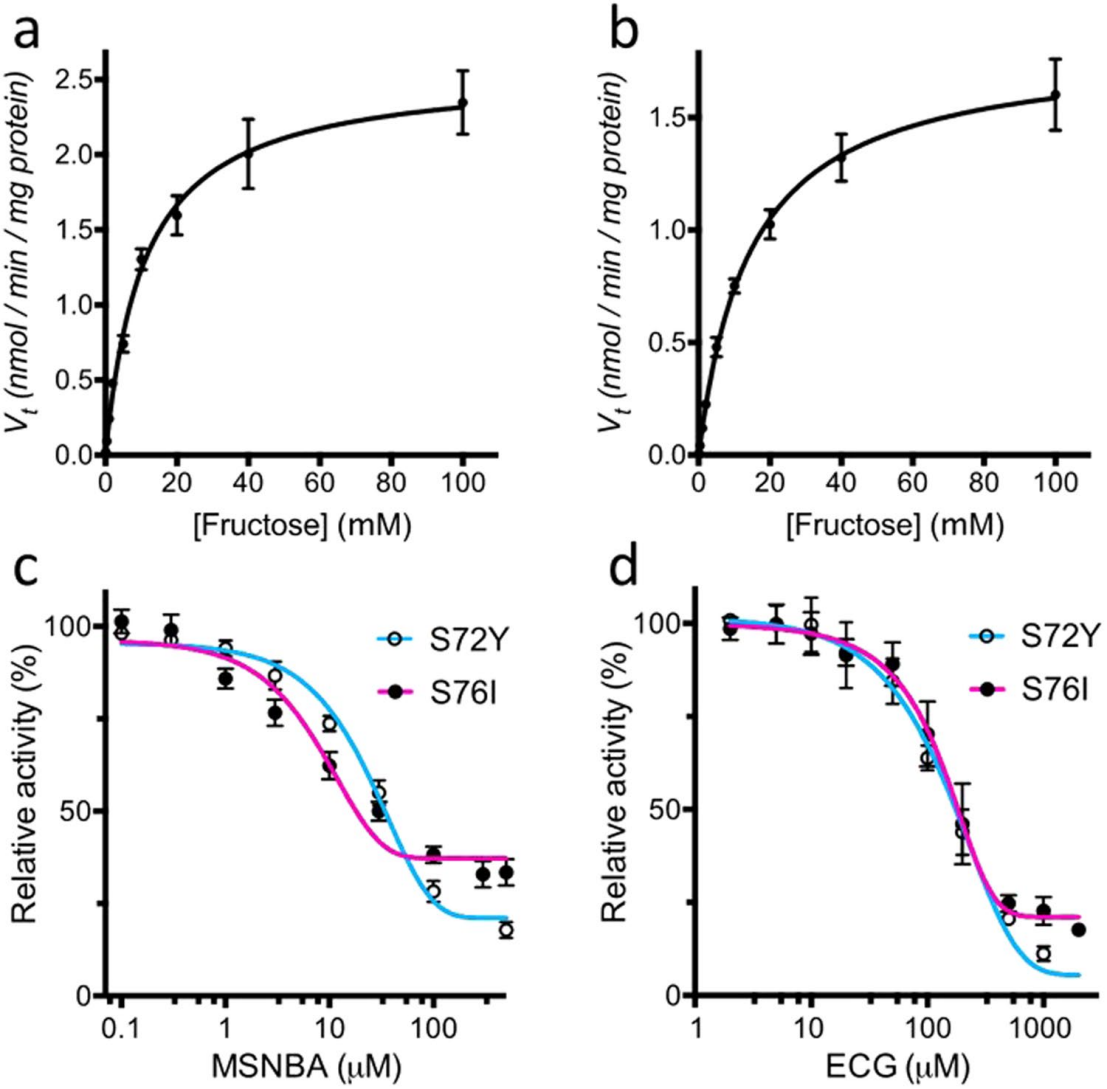
Transport kinetics and inhibition of GLUT5 mutants in *hxt* ^*0*^ cells. The initial uptake velocity v_*t*_ (expressed as nmol fructose transported per minute per mg cell dry weight) was measured for GLUT5tr^S72Y^ (**a**) and GLUT5tr^S76I^ (**b**) and plotted against sugar concentration. Mean values and standard deviation were calculated from biological triplicates. The lines represent a least-square fit to the Michaelis-Menten equation. The uptake of fructose was measured at variable MSNBA (**c**) and ECG (**d**) concentrations and the uptake velocity was normalized to that in the absence of the inhibitor. The IC_50_ values were calculated by nonlinear algorithm plots supplied by Prism (GraphPad Software).

Further, we investigated whether the mutations have altered the properties of GLUT5 concerning transport kinetics and action of inhibitors. Recently, MSNBA was identified as a potent and specific inhibitor of GLUT5^20^. To test the inhibition of yeast-expressed GLUT5, variable concentrations (1–50 µm) of MSNBA were added to YEPF media and the growth of the transformants was monitored. As expected, the growth of the cells expressing GLUT5tr^S72Y^ and GLUT5tr^S76I^ was delayed by MSNBA in a concentration dependent manner, while there was virtually no influence on cells expressing *HXT7* (compare Fig. 3b–d). This observation confirms a GLUT5-specific inhibition mechanism. To further corroborate that mutated GLUT5 in yeast behaves like the WT protein in animal cells, we performed a similar assay with ECG, an inhibitor that is structurally not related to MSNBA. Again, the growth of the *hxt* ^*0*^ cells expressing GLUT5 variants was delayed by the inhibitor, while the *HXT7* expressing control was not affected (Supplementary Fig. S7). For a quantitative insight into transport kinetics of GLUT5 mutants, we determined the Michaelis-constant (K_M_ values) for fructose (Fig. 4a,b and Table 2) and half maximal inhibitory concentration (IC_50_ values) of MSNBA and ECG (Fig. 4c,d and Table 2). Although there is a slight variation between the mutants, their kinetic parameters are similar to those determined in MCF-7 cells^17, 20^ or *X*. *laevis* oocytes^18^, indicating that the mutations do not have a major influence on the transport mechanism. Reported K_M_ values for human GLUT5 fructose uptake vary between 6 and 15 mM^3, 17, 40^.

**Table 2:** GLUT5 transport kinetics and inhibition in *hxt* ^*0*^ yeast cells (see also Fig. 4).

GLUT5 variant	K_M_ [mM]	IC_50_ of MSNBA [µM]	IC_50_ of ECG [µM]
S72Y	10.8 ± 1.0	26.6 ± 6.7	180 ± 11
S76I	14.6 ± 1.1	6.3 ± 0.7	140 ± 11
WT in MCF-7 cells or in *X*. *laevis* oocytes*	~10^17^*	5.8 ± 0.5^20^*	K_i_ = 117 ± 54^18^*

*Values from References indicated in superscript.

## Discussion

In this work, we successfully complemented the hexose transporter-deficient strain EBY.VW4000 with the human fructose transporter GLUT5. The wild-type protein was not active in the *hxt* ^*0*^ strain or even in its derivative EBY.S7 carrying the *fgy1* mutation that promoted the activity of GLUT1^32^. However, several clones that carried random, PCR-born mutations in the GLUT5 ORF could be identified, which were capable of fructose transport. Interestingly, in all clones serine residues (at positions 72 and 76) were mutated. They are located in the same region of the protein as mutations previously found to be required for GLUT1 activity in *hxt* ^*0*^ strains (Supplementary Fig. S5). The mutations appeared to have no influence on the subcellular distribution of the protein (Fig. 1b), similar to what was observed with GLUT1 previously^32^. Thus, it is possible that the mutations affect the conformational dynamics of the transporter in the non-native lipid environment. For instance, GLUT3 and GLUT4 transport activities depend on the type of lipids used for proteoliposome generation, with anionic phospholipids playing a significant role in stabilizing the transporters and conical lipids enhancing transport activity^41^. Strikingly, the mutation *fgy4*, that is required for GLUT4 functionality in the *hxt* ^*0*^ strain^32^, prevents the last step of ergosterol biosynthesis (i.e. the mutants accumulate ergosta-5,7,22,24(28)-tetraen-3β-ol in the plasma membrane; E. Boles, unpublished result). This further underscores the impact of protein-lipid interactions.

Interestingly, both mutations in TM2 (S72Y and S76I) seem to favor the outward-facing conformation; the residue substitutions require TM2 and 11 to move apart by 1–2 Å in the inward-facing conformation models (Table 1 and Fig. 2). Analysis of GLUT sequences shows that positions 72 and 76 are generally occupied by small side-chains. The substitutions of S76 with a branched hydrophobic residue or of S72 with a bulky Tyrosine, impacts the inward-facing conformation in which there is close juxtaposition of TM2 and TM11. Thus, we speculate that the mutations in TM2 destabilize the inward-facing conformation relative to the outward-facing conformation of GLUT5. It is possible that favoring the outward-facing conformation of the transporter compensates for alterations in the conformational dynamics of the transporter in the non-native lipid milieu and this confers an advantage to these GLUT5 mutants over the wild-type with respect to fructose transport by reducing the overall length of the catalytic cycle. Regardless of the mechanism underlying the effect of the mutations, it is important for screening approaches that they do not have a major influence on transport kinetics and inhibition. Indeed we show here that the K_M_ of fructose uptake by GLUT5 mutants in yeast *hxt* ^*0*^ cells (abbreviated *hxt* ^*0*^_GLUT5_; Table 2) is comparable with that determined in other systems used to assay GLUT5)^3, 17, 40^. Additionally, the effects of reported GLUT5 inhibitors on fructose uptake in the *hxt* ^*0*^_GLUT5_ were similar to previous results in other GLUT5 systems including human cancer cell line (MCF-7) and GLUT5 expressed in *X*. *laevis* oocytes. Thus, with the *hxt* ^*0*^_GLUT5_ system IC_50_ value for fructose uptake inhibition by (−)-epicatechin-gallate (ECG) was comparable to the K_i_ value previously reported^18^. Also the IC_50_ for MSNBA inhibition of fructose uptake by *hxt* ^*0*^_GLUT5_ is comparable to that determined in human breast carcinoma MCF-7 cells and ~10-fold lower than that determined in GLUT5 proteoliposomes^20^, making the yeast system superior to GLUT5 proteoliposomes and comparable to human cell lines systems in assessing GLUT5 inhibition.

Importantly, inhibition of GLUT5 transport by ECG and MSNBA evidenced in cell-growth supports the use of *hxt* ^*0*^_GLUT5_ system for facile, low-cost and high-throughput screening of GLUT5 inhibitors. High-throughput screening of GLUT5 ligands by *hxt* ^*0*^ yeast cell-growth is currently being implemented in our laboratories. Furthermore, *hxt* ^*0*^_GLUT5_ system does not need to account for complicating contributions to the fructose transport from other GLUTs or carbohydrate transporters, as is the case in animal cell lines. Compared to alternative yeast strains, e.g. RE700A, which lacks only the major hexose transporters HXT1-HXT7^38^ and which was successfully used to express and characterize human GLUT1^42^, the *hxt* ^*0*^ strain EBY.VW4000 offers the advantage that no residual hexose transporters are left in its genome. Many of the remaining hexose transporters in RE700A, e.g. Hxt11 or Gal2 are repressed on glucose, but can be upregulated under selective pressure^26^, which could likely lead to a high frequency of suppressor mutations and therefore severely complicate high-throughput screening approaches. Thus, despite the fact that several genomic alterations have occurred in the *hxt* ^*0*^ strain due to the repeated use of the loxP-Cre recombinase system^43^, it has remained the most popular and widely used system for characterization of heterologous hexose transporters. While GLUT5 proteoliposome system is also hexose transporter-free, it is more labor-intensive than *hxt* ^*0*^_GLUT5_. Overall, *hxt* ^*0*^_GLUT5_ is a convenient, versatile, low-cost, GLUT5-specific, and fast system for identification and characterization of human GLUT5 ligands, allowing the screening of compound libraries for potential GLUT5 effectors that could serve as drugs for cancer, metabolic disease, and diabetes. Using a growth-based, microbial screening system will dramatically accelerate the cycle of inhibitor discovery and characterization.

## Methods

### Yeast strains, media and growth conditions

Construction of the strain EBY.VW4000 was described previously^26^ and the relevant genotype is shown in Supplementary Table S1. Plasmid-free cells were grown in standard YEP-media (1%(w/v) yeast extract, 2%(w/v) peptone) supplemented with 1% (w/v) maltose for maintenance and preparation of competent cells. Frozen competent cells were prepared and transformed according to the established protocol^44^. The synthetic complete (SC) medium^39^ was supplemented with the appropriate carbon source (as indicated in the figure legends), whereby Uracil was dropped out (-Ura) for plasmid selection and Methionine was omitted to induce the expression from the *MET25* promoter, where indicated. For selection of plasmids containing the *kanMX4* cassette, 200 µg/ml G-418 was added to the YEP-based media.

### PCR and plasmid construction

Codon optimized GLUT5 was synthesized by GenScript. The sequences are listed in Supplementary Table S3. The primers used for ORF amplification are listed in Supplementary Table S2. The PCR reactions (50 µl final volume) contained 10 ng of template DNA, 500 nM of each primer, 0.2 mM of deoxy-nucleotides, and 1 Unit of the Phusion polymerase in 1x concentration of the supplied HF-buffer (Thermo Fisher). After initial denaturation (2 min at 98 °C), 28 cycles were performed (15 s at 98 °C; 30 s at 58 °C; 45 s at 72 °C). The PCR products (500 ng) were co-transformed with 800 ng of BamHI/XhoI linearized p426MET25 vector into frozen competent cells of EBY.VW4000 to allow for plasmid assembly by homologous recombination (gap-repair procedure)^34^. The transformants were plated on SC-Ura media with 1% (w/v) maltose (SCM-Ura). After two days of incubation at 30 °C, the colonies were replica-plated onto SC-Ura media containing 2% (w/v) fructose (SCF-Ura). The resulting colonies were subcultivated and the plasmid DNA was isolated by the standard alkaline lysis protocol and transferred into *E*. *coli* DH10B cells by electroporation. The plasmid DNA was isolated from *E*. *coli* overnight cultures using a commercial kit according to manufacturer’s recommendations and sequenced at GATC Biotech (Konstanz, Germany). After the mutations in the GLUT5 sequence were isolated, the ORFs from the p426MET25-based plasmids were amplified as described above and inserted into the pRS72K vector between the truncated *HXT7* promoter and *CYC1* terminator by the gap-repair procedure. sGFP was inserted by the gap-repair method into p426MET25-based GLUT5 constructs, which were linearized with XhoI between the GLUT5 ORF and the *CYC1* terminator. The relevant plasmid properties are listed in Supplementary Table S4.

### Growth tests on solid and liquid media

For growth tests on agar plates, cells were pre-grown on selective maltose-containing medium and washed two times with sterile water. The cell density was adjusted to OD_600nm_ = 1. Five microliters of the cell suspension were then spotted onto appropriate agar plates, which were incubated at 30 °C for time periods indicated in the figure legends.

The growth kinetics in liquid cultures was measured with the Cell Growth Quantifier (CGQ; Aquila Biolabs). Precultures were grown overnight in YEP containing 1% (w/v) maltose and 200 µg/ml G-418 for plasmid selection. The cells were washed twice with sterile water and resuspended to a final OD_600nm_ = 0.5 in YEP medium containing 1% (w/v) fructose and 200 µg/ml G-418. The cultures were filled into 300 ml shake flasks and mounted onto the CGQ sensor plate. The measurement was performed as previously described^39^.

### Fluorescence microscopy

For the *in vivo* investigation of protein localization, EBY.VW4000 cells expressing the indicated constructs were grown in selective SCM-Ura media without Methionine until an OD_600nm_ = 2.0–2.5 was reached. Cells were harvested by centrifugation at 3,000 × g for 1 min and resuspended in the same media containing 0.3% (w/v) low melting agarose for immobilization. Ten microliters were applied to an object plate, sealed with a cover slip, and analyzed with a Confocal Laser Scanning Microscope (TCS SP5 378, Leica Microsystems AG, Wetzlar, Germany) for GFP fluorescence.

### Sugar uptake and inhibition assays

GLUT5 (GLUT5tr^S72Y^ or GLUT5tr^S76I^) expressing EBY.VW4000 yeast strains were grown in 10 ml of YEPM media with 100 µg/L antibiotic G-418, at 29 °C, in an incubator with shaking (220 rpm) for one day. Two mililiters of cell culture were centrifuged at 10,000 g for 30 seconds and the pellet was resuspended in 50 ml of YEPF media with 100 µg/L G-418. The cells were grown as above to OD_600nm_ ~ 10 (for 1–2 days), then harvested by centrifugation at 5,000 g for 2 minutes. The cell pellet was resuspended in 50 ml PBS buffer and centrifuged once more. The pellet was resuspended in PBS buffer at OD_600nm_~1.5. Cells that did not express GLUT5 were used as background control. Transport assay was initiated at 22 °C by the addition of 2 μl of ^14^C-radiolabeled fructose (50 nCi, 0.2 nmol) and various concentrations of cold-fructose to 100 or 200 μl yeast cell solution with/without the inhibitor N-[4-(methylsulfonyl)-2-nitrophenyl]-1,3-benzodioxol-5-amine (MSNBA, Enamine) or (−)-epicatechin-gallate (ECG, Cayman). The transport was stopped after 20 minutes with ice-chilled quench buffer (0.1 M KPi, pH 5.5, 0.1 M LiCl), the solution was filtered with 0.4 µm cellulose nitrate membrane filter (Whatman), and the filter was washed twice with the quench buffer. The membrane filter was placed into a vial filled with BioSafe II scintillation liquid (Research Products International Corp.) and radioactivity was measured with Becker LS 6500 Multi-purpose Scintillation Counter. Kinetic parameters were determined by non-linear algorithm plots supplied by Prism (GraphPad Software).

### Modeling of GLUT5^S76I^ and GLUT5^S72Y^ structures

GLUT5 model structures of inward- and outward-facing conformations were generated with COOT^45^ on the basis of crystal structure of bovine GLUT5 (PDB ID 4YB9) for the inward-facing conformation and mouse GLUT5 (PDB ID 4YBQ) for the outward-facing conformation^37^. Energy minimization of mutant structures was performed with Phenix^46^.

## Electronic supplementary material

Supplementary Information
