# Supplementary material for: Establishing a yeast-based screening system for discovery of human GLUT5 inhibitors and activators
Source: Sci Rep. 2017 Jul 24;7:6197. doi: 10.1038/s41598-017-06262-4 (PMC5524692; doi:10.1038/s41598-017-06262-4)
Supplement: Supplementary file 1 — Supplementary Information [file 41598_2017_6262_MOESM1_ESM.pdf]

Supplementary Information

Establishing a yeast-based screening system for discovery of human GLUT5 inhibitors and activators

Joanna Tripp, Christine Essl, Cristina Iancu, Eckhard Boles, Jun-yong Choe and Mislav Oreb

Supplementary Table S1: Genotype of the *hxt^0* strain

Strain name	Relevant genotype
EBY.VW4000	*MATa leu2-3,112 ura3-52 trp1-289 his3-1 MAL2-8c SUC2 Δhxt1-17 Δgal2 Δstl1::loxP Δagt1::loxP Δmph2::loxP Δmph3::loxP*

Supplementary Table S2: Primers used in this study

**Template DNA**	**Primer Name**	**Sequence (5' - 3')**	**Application**
	MOP329	GATACAATTCTATTACCCCCATCCATACTCTAGAAATGAAAGAAGGTCGTCTGAC	forward primer for gap-repair cloning into p426MET25
	MOP330	ATGTAAGCGTGACATAACTAATTACATGACTCGAGTTATTGTTCAGAGGTCACCG	reverse primer for gap-repair cloning into p426MET25 and pRS72K
	MOP331	ATTACCCCCATCCATACTCTAGAAATGGAACAACAAGACCAATCTATGAAAGAAGGTCGTCTGAC	forward primer for introduction of 7 N-terminal amino acids into GLUT5tr and gap-repair cloning into p426MET25
GLUT5tr	MOP370	ACAAAAACAAAAAGTTTTTTTAATTTTAATCAAAAAATGAAAGAAGGTCGTCTGAC	forward primer for gap-repair cloning into pRS72K
	MOP441	CTGAAAGAACTGCCGCCGGTGACCTCTGAACAAAGTAAAGGAGAAGAACTTTTCACTG	forward primer for fusion of sGFP with the C-terminus of GLUT5tr variants
sGFP	JTP97	AATGTAAGCGTGACATAACTAATTACATGATTATTTGTAGAGCTCATCCATGC	reverse primer for gap-repair cloning into p426MET25

Supplementary Table S3: ORF sequences of GLUT5 variants

GLUT5 variant	ORF sequence
GLUT5tr	ATGAAAGAAGGTCGTCTGACCCTGGTTCTGGCTCTGGCTACCCTGATCGCCGCCTTCGGT<br>TCTTCTTTCCAATACGGTTACAACGTGGCTGCTGTTAACAGCCCGGCTCTGCTGATGCAA<br>CAGTTCTATAACGAGACCTATTACGGTCGCACCGGTGAGTTCATGGAAGATTTCCCACTG<br>ACCCTGCTGTGGTCTGTTACTGTTAGCATGTTCCCGTTCGGCGGTTTCATTGGTAGCCTG<br>CTGGTTGGTCCACTGGTGAACAAGTTCGGTCGCAAGGGTGCTCTGCTGTTCAACAACATT<br>TTCAGCATTGTTCCGGCTATTCTGATGGGTTGTTCTCGCGTTGCTACCTCCTTCGAGCTG<br>ATCATTATTTCTCGTCTGCTGGTTGGTATTTGCGCCGGCGTCAGCAGCAACGTTGTGCCA<br>ATGTATCTGGGTGAACTGGCTCCAAAGAACCTGCGTGGTGCTCTGGGTGTTGTTCCACAG<br>CTGTTCATCACCGTTGGCATTCTGGTTGCTCAGATTTTCGGTCTGCGTAACCTGCTGGCT<br>AACGTTGACGGTTGGCCAATCCTGCTGGGTCTGACTGGTGTTCCAGCTGCTCTGCAACTG<br>CTGCTGCTGCCATTCTTCCCGGAATCTCCGCGTTACCTGCTGATCCAGAAAAAAGACGAA<br>GCCGCCGCCAAGAAAGCTCTGCAGACTCTGCGTGGTTGGGATTCCGTTGACCGTGAAGTT<br>GCTGAAATTCGCCAGGAAGATGAAGCTGAAAAAGCTGCTGGTTTCATCTCTGTTCTGAAG<br>CTGTTCCGTATGCGTAGCCTGCGTTGGCAGCTGCTGAGCATCATCGTTCTGATGGGTGGT<br>CAACAGCTGAGCGGTGTTAACGCTATCTACTATTATGCTGATCAGATCTACCTGTCTGCT<br>GGCGTTCCGGAAGAGCATGTCCAGTATGTTACCGCTGGCACTGGTGCTGTTAACGTTGTT<br>ATGACTTTCTGCGCTGTTTTCGTTGTCGAACTGCTGGGTCGTCGTCTGCTGCTGCTGCTG<br>GGTTTCTCTATCTGCCTGATCGCTTGCTGCGTTCTGACTGCTGCTCTGGCTCTGCAGGAT<br>ACCGTTTCTTGGATGCCGTATATTTCTATCGTTTGCGTGATTTCTTACGTTATCGGTCAC<br>GCTCTGGGTCCAAGCCCGATCCCAGCTCTGCTGATCACCGAGATCTTCCTGCAGTCTAGC<br>CGTCCGAGCGCTTTCATGGTTGGTGGTTCTGTTCACTGGCTGTCTAACTTCACCGTTGGT<br>CTGATCTTCCCGTTCATCCAGGAAGGTCTGGGTCCATATTCCTTCATCGTGTTCGCCGTT<br>ATCTGTCTGCTGACCACCATTTACATCTTCCTGATCGTGCCAGAGACCAAGGCCAAGACC<br>TTCATCGAGATCAACCAAATCTTCACCAAGATGAACAAAGTGAGCGAGGTTTACCCGGAA<br>AAAGAGGAGCTGAAAGAACTGCCGCCGGTGACCTCTGAACAATAA
GLUT5	ATGGAACAACAAGACCAATCTATGAAAGAAGGTCGTCTGACCCTGGTTCTGGCTCTGGCT<br>ACCCTGATCGCCGCCTTCGGTTCTTCTTTCCAATACGGTTACAACGTGGCTGCTGTTAAC<br>AGCCCGGCTCTGCTGATGCAACAGTTCTATAACGAGACCTATTACGGTCGCACCGGTGAG<br>TTCATGGAAGATTTCCCACTGACCCTGCTGTGGTCTGTTACTGTTAGCATGTTCCCGTTC<br>GGCGGTTTCATTGGTAGCCTGCTGGTTGGTCCACTGGTGAACAAGTTCGGTCGCAAGGGT<br>GCTCTGCTGTTCAACAACATTTTCAGCATTGTTCCGGCTATTCTGATGGGTTGTTCTCGC<br>GTTGCTACCTCCTTCGAGCTGATCATTATTTCTCGTCTGCTGGTTGGTATTTGCGCCGGC<br>GTCAGCAGCAACGTTGTGCCAATGTATCTGGGTGAACTGGCTCCAAAGAACCTGCGTGGT<br>GCTCTGGGTGTTGTTCCACAGCTGTTCATCACCGTTGGCATTCTGGTTGCTCAGATTTTC<br>GGTCTGCGTAACCTGCTGGCTAACGTTGACGGTTGGCCAATCCTGCTGGGTCTGACTGGT<br>GTTCCAGCTGCTCTGCAACTGCTGCTGCTGCCATTCTTCCCGGAATCTCCGCGTTACCTG<br>CTGATCCAGAAAAAAGACGAAGCCGCCGCCAAGAAAGCTCTGCAGACTCTGCGTGGTTGG<br>GATTCCGTTGACCGTGAAGTTGCTGAAATTCGCCAGGAAGATGAAGCTGAAAAAGCTGCT<br>GGTTTCATCTCTGTTCTGAAGCTGTTCCGTATGCGTAGCCTGCGTTGGCAGCTGCTGAGC<br>ATCATCGTTCTGATGGGTGGTCAACAGCTGAGCGGTGTTAACGCTATCTACTATTATGCT<br>GATCAGATCTACCTGTCTGCTGGCGTTCCGGAAGAGCATGTCCAGTATGTTACCGCTGGC<br>ACTGGTGCTGTTAACGTTGTTATGACTTTCTGCGCTGTTTTCGTTGTCGAACTGCTGGGT<br>CGTCGTCTGCTGCTGCTGCTGGGTTTCTCTATCTGCCTGATCGCTTGCTGCGTTCTGACT<br>GCTGCTCTGGCTCTGCAGGATACCGTTTCTTGGATGCCGTATATTTCTATCGTTTGCGTG<br>ATTTCTTACGTTATCGGTCACGCTCTGGGTCCAAGCCCGATCCCAGCTCTGCTGATCACC<br>GAGATCTTCCTGCAGTCTAGCCGTCCGAGCGCTTTCATGGTTGGTGGTTCTGTTCACTGG<br>CTGTCTAACTTCACCGTTGGTCTGATCTTCCCGTTCATCCAGGAAGGTCTGGGTCCATAT<br>TCCTTCATCGTGTTCGCCGTTATCTGTCTGCTGACCACCATTTACATCTTCCTGATCGTG<br>CCAGAGACCAAGGCCAAGACCTTCATCGAGATCAACCAAATCTTCACCAAGATGAACAAA<br>GTGAGCGAGGTTTACCCGGAAAAAGAGGAGCTGAAAGAACTGCCGCCGGTGACCTCTGAA<br>CAATAA

Supplementary Table S4: Plasmids used in this study

Plasmid name	Relevant properties and references
p426MET25	2µ origin; *URA3* marker; methionine-repressible MET25 promoter[1]
pRS72K	2µ origin; *TEF* promoter controlling *kanMX4* was exchanged by *TDH3* promoter in the pRS42K[2] backbone. A cassette comprising truncated *HXT7* promoter, multiple cloning site and *CYC1* terminator was integrated for heterologous gene expression. This cassette was amplified from the p426HXT7 vector[3]

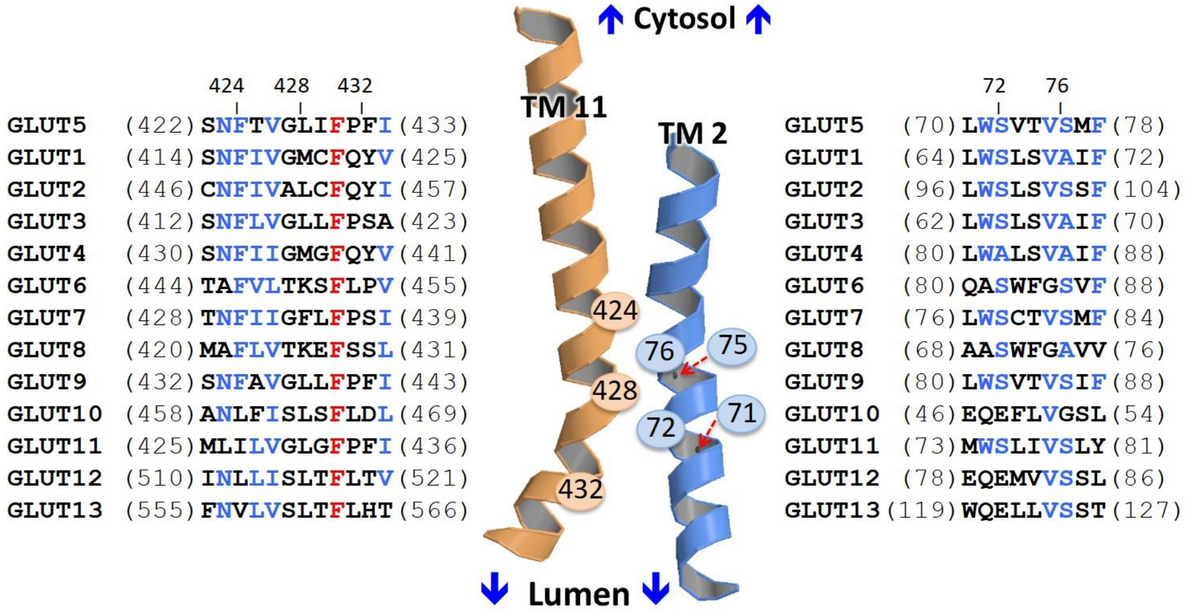

Supplementary Figure S5: Localization of mutations in the TM domains of GLUT5. Shown is an alignment of relevant amino acid sequences (TM11, left and TM2, right) of GLUT1-GLUT13. The degree of conservation is indicated by the color code (none, black; moderate, blue; strict, red). The residues, which were mutated in yeast-expressed transporters, are S72, S76 of GLUT5 and W65, V69 of GLUT1. The positions of S72 and S76 as well those of interacting residues in TM11 (F424, L428 and F432) are shown in a model of TM11 and TM2 of GLUT5.

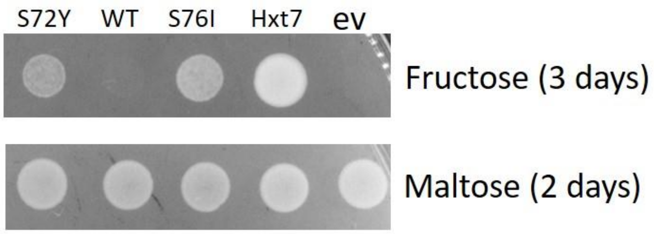

Supplementary Figure S6: Growth of EBY.VW4000 expressing GLUT5 variants on fructose and maltose. Serial dilutions of cells transformed with plasmids encoding GLUT5 variants (wild-type GLUT5tr; GLUT5tr^{S76I}; GLUT5tr^{S72YT}) were dropped onto indicated media. Empty vector (ev) was used as a negative control and a plasmid encoding the endogenous high-affinity hexose transporter Hxt7 as a positive control for growth on fructose. Maltose is shown as a viability control of the transformants. The plates were incubated at 30°C for two or three days.

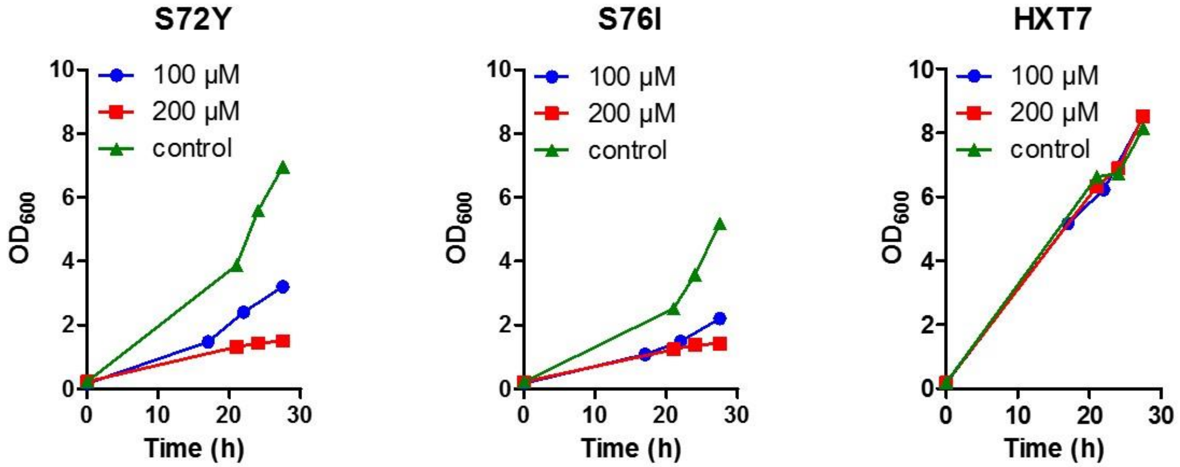

Supplementary Figure S7: Inhibition of GLUT5 expressed in yeast cells by ECG. The EBY.VW4000 cells transformed with plasmids encoding GLUT5tr^{S72Y}, GLUT5tr^{S76I} or Hxt7 were cultivated in YEP media containing 2% (w/v) fructose and 200 µg/ml of G418 for plasmid selection. ECG was added at indicated concentrations or omitted (control). The growth was monitored over time by measuring OD_{600nm} of the culture. The results represent one measurement.

Supplementary References

1. Mumberg, D., Müller, R. & Funk, M. Regulatable promoters of Saccharomyces cerevisiae: comparison of transcriptional activity and their use for heterologous expression. *Nucleic Acids Res.* **22,** 5767–5768 (1994).
2. Taxis, C. & Knop, M. System of centromeric, episomal, and integrative vectors based on drug resistance markers for Saccharomyces cerevisiae. *BioTechniques* **40,** 73–78 (2006).
3. Hamacher, T., Becker, J., Gardonyi, M., Hahn-Hagerdal, B. & Boles, E. Characterization of the xylose-transporting properties of yeast hexose transporters and their influence on xylose utilization. *Microbiology* **148,** 2783–2788 (2002).
